# A TAT Peptide-Functionalized Liposome Delivery Phage System (TAT-Lip@PHM) for an Enhanced Eradication of Intracellular MRSA

**DOI:** 10.3390/pharmaceutics17060743

**Published:** 2025-06-05

**Authors:** Kaixin Liu, Xin Lu, Xudong Guo, Yi Yang, Wanying Liu, Hongbin Song, Rongtao Zhao

**Affiliations:** 1 Chinese PLA Center for Disease Control and Prevention, Beijing 100071, China; 15890884064@163.com (K.L.);; 2 The Fifth Medical Center of Chinese PLA General Hospital, Beijing 100071, China

**Keywords:** intracellular MRSA, phage delivery, TAT peptide, liposome

## Abstract

**Background:** Intracellular bacteria frequently result in chronic and recurrent infections. MRSA is one of the most prevalent facultative intracellular bacteria in clinical infections. The drug resistance of MRSA and the difficulty of most antibiotics in entering cells result in a suboptimal clinical efficacy of antibiotics in the treatment of intracellular MRSA. Bacteriophages represent a promising alternative therapy in the context of the current antimicrobial resistance crisis. Nevertheless, the low efficiency of phage entry into cells and their rapid inactivation remain challenges in the treatment of intracellular MRSA using phages. The utilization of functionalized carriers for the delivery of phages into cells and their protection represents a feasible strategy. **Methods:** In this study, a new MRSA bacteriophage (vB_SauS_PHM) was isolated from hospital sewage, exhibiting the characteristics of short incubation period, large lytic amount, and good environmental tolerance. Subsequently, vB_SauS_PHM was encapsulated by TAT peptide-functionalized liposomes through microfluidic technology and size-exclusion chromatography (SEC), forming a phage delivery system, designated TAT-Lip@PHM. **Results:** The encapsulation rate of the phage by TAT-Lip@PHM was 20.3%, and the cell entry efficiency was ≥90% after 8 h. The 24 h eradication rate of 300 μg/mL TAT-Lip@PHM against intracellular MRSA was 94.05% (superior to the 21.24% and 44.90% of vB_SauS_PHM and Lip@PHM, respectively), while the mammalian cell activity was >85% after 24 h incubation. **Conclusions:** The TAT-Lip@PHM effectively delivered the phage into the cell and showed an excellent killing effect on intracellular MRSA with low cytotoxicity. This work provides a technical reference for the application of phages in the treatment of intracellular bacterial infection.

## 1. Introduction

Intracellular bacteria are bacteria that are capable of surviving and reproducing within host cells following invasion of the body through the skin, mucosa, respiratory tract, or other routes. They possess the ability to evade the host immune system and to kill antibiotics, frequently resulting in chronic and recurrent infections [1]. *S. aureus* is a facultative intracellular bacterium that causes a wide range of nosocomial and community-acquired infections. *S. aureus* can enter various cells, such as keratinocytes and endothelial cells, through internalization, and it can even invade phagocytic cells that are responsible for the immune clearance of bacteria, resulting in persistent intracellular infection [2,3]. Methicillin-resistant *S. aureus* (MRSA) represents the predominant antibiotic-resistant strain of *S. aureus*. In comparison to methicillin-susceptible *S. aureus* (MSSA), MRSA has been demonstrated to result in a higher incidence of morbidity and mortality [4]. MRSA has demonstrated resistance to a range of antibiotics, resulting in over 100,000 deaths globally in 2019 [5]. At present, approximately 30% of nosocomial infections are attributed to MRSA. The multi-drug resistance (MDR) of MRSA, combined with the protective barrier of host cells, makes it difficult for antibiotics to kill intracellular MRSA, which has become a serious threat to public health [6].

A bacteriophage/phage is a type of virus that can infect and lyse bacteria [7]. In recent years, with the increasing prevalence of antibiotic resistance, bacteriophages, as non-antibiotic bactericidal substances, have attracted increasing attention [8,9,10]. Studies on the use of phages against extracellular MRSA have been widely reported. For example, Simon et al. [11] employed phage Sb-1 to reduce MRSA by 35% in vitro and ∼90% when combined with oxacillin. Teng Fei et al. [12] utilized phage 4086-1 to reduce MRSA in mouse mammary glands by 8 log_10_ CFU/g and to mitigate the inflammatory response in mouse mammary glands. Furthermore, phage therapy also demonstrated favorable efficacy and safety outcomes in the treatment of MRSA-induced prosthetic knee infections [13,14]. However, the utilization of phages to eradicate intracellular bacteria remains a challenging endeavor, necessitating technological advancement. Single-ingredient phages are liable to be identified as foreign antigens and rapidly eliminated by the immune system, and it is difficult for phages to invade eukaryotic cells to eliminate intracellular bacteria [15].

The development of carriers that can effectively protect phages and deliver them into cells is a promising strategy to improve the killing ability of phages against intracellular bacteria. A liposome is a miniature spherical carrier with a bilayer structure formed by phosphatidylcholine and cholesterol. Liposomes can encapsulate drugs, phages, etc., in their lipid bilayers and then deliver the “cargo” into the cell through membrane fusion or internalization [16,17]. Liposomes have good biocompatibility and sustained release and could enhance the stability of encapsulated drugs. Currently, dozens of liposomal drug-delivery systems have been approved by the FDA [18].

However, liposomes prepared by traditional methods, such as electroformation, thin-film hydration, sonication, solvent dispersion, reverse-phase evaporation, and thin-film extrusion, have the disadvantages of small particle size and inhomogeneity and are unable to encapsulate large-size (>300 nm) phages [19]. The newly developed microfluidic controlled preparation method has the advantages of small reagent consumption and a controllable average size of liposomes. By optimizing the material ratio and production parameters, stable and uniform large unilamellar vesicles (0.1–1 μm) can be prepared to achieve the encapsulation of large-size phages [18,20].

In addition, most drug carriers undergo lysosomal transport after uptake by cells, during which bacteriophages are prone to be inactivated by enzymes, resulting in a decreased intracellular concentration and bactericidal potency. In 1988, Frankel et al. [21] found that one transactivator of human immunodeficiency virus could penetrate the cell membrane and enter the cell; it was later called TAT cell-penetrating peptide (CPP). Due to its good transmembrane activity and low cytotoxicity [22], TAT peptide has been widely applied to deliver nanoparticles, proteins, nucleic acids, and small-molecule drugs into cells to enhance the intracellular biological effects of these substances [23,24]. Functionalizing drug carriers with CCPs to improve their ability to directly penetrate the cell membrane is a feasible strategy to reduce phage enzymatic hydrolysis [25,26].

In this study, a novel bacteriophage that can lyse MRSA (named vB_SauS_PHM) was isolated from hospital sewage. Then, a phage-encapsulated TAT liposome delivery system (TAT-Lip@PHM) was constructed using microfluidic technology and a TAT modification. TAT-Lip@PHM effectively delivered vB_SauS_PHM into cells and showed a good bactericidal effect against intracellular MRSA. The intended route of administration of TAT-Lip@PHM is intravenous injection, aiming at systemic intracellular MRSA infection. It is hoped that this study can provide a technical reference for the use of phages to treat intracellular MRSA infection.

## 2. Materials and Methods

### 2.1. Strains and Samples

Medical sewage samples were collected from a hospital in Beijing (permission was obtained from the hospital) for phage isolation. MRSA strains stored in our laboratory were isolated from hospital clinical samples. One strain, named MRSA 1, was used as the host bacteria for phage purification and biological characterization. RAW 264.7 mouse mononuclear macrophages were purchased from Beijing Zoman Biotechnology Co., Ltd. (Beijing, China). A green fluorescent-labeled MRSA strain (USA 300) was purchased from Guangzhou Changyan Biotechnology Co., Ltd. (Guangzhou, China).

### 2.2. Reagents and Instruments

PEG8000, Triton X-100, and an MTT cell proliferation and cytotoxicity detection kit were purchased from Solarbio Science & Technology Co., Ltd. (Beijing, China); DNase I and RNase were purchased from Winter song Boye Biotechnology Co., Ltd. (Beijing, China); Mung Bean Nuclease was purchased from TaKaRa (Luhe tong Economic & Trading Co., Ltd., Beijing, China); DMEM medium, fetal bovine serum, and penicillin–streptomycin were purchased from Gibco (Tianguang Baiquan Technology Co., Ltd., Beijing, China); 1,2-distearoyl-sn-glycero-3-phosphocholine (DSPC), DSPE-PEG2000, and Cy5 were purchased from Aladdin Corporation (Shanghai, China); and a SuperEV ultrapure size-exclusion chromatography column was purchased from Rengen Biosciences (Yingze Tonghui Biology Science and Technology Co., Ltd., Beijing, China).

A constant-temperature oscillating shaker was purchased from Saiouhuachuang technology co., Ltd. (Beijing, China); a cryogenic centrifuge was purchased from Sartorius Lab Instruments GmbH & Co. KG (37070 Goettingen, Germany); a multifunctional microplate reader was purchased from Molecular Devices (Shanghai, China); a gel imager was purchased from Tanon Corporation (Shanghai, China); an automatic cell counter was purchased from Thermo Fisher Scientific (Life Technologies Holdings Pte Ltd., Singapore); microfluidics equipment was purchased from Mingtai pharmaceutical Equipment Co., Ltd. (Shanghai, China); and a microfluidic chip was designed and fabricated by the Institute of Electronics, Chinese Academy of Sciences. A nanoparticle size and Zeta potential analyzer was purchased from Malvern Panalytical company; a laser confocal microscope was purchased from OLYMPUS Corporation (Tokyo 163-0914, Japan).

### 2.3. Isolation and Purification of Phage

The sampled hospital sewage was centrifuged with the cryogenic centrifuge (Sartorius G-26C, 37070 Goettingen, Germany) at 6656× *g* for 10 min, and the supernatant was filtered through a 0.22 μm filter to obtain the crude phage treatment solution. A total of 1 mL logarithmic-phase host bacteria and 1 mL phage crude treatment solution were incubated in 10 mL LB medium at 37 °C for 8 h. The phage stock solution was obtained using the cryogenic centrifuge (Sartorius G-26C, 37070 Goettingen, Germany) at 6656× *g* for 10 min, followed by filtration of the supernatant; this was repeated three times. In the double agar overlay drop plaque assay, 100 μL logarithmic phase host bacteria and 5 mL melted semi-solid medium were mixed and poured into the bottom agar plate. After coagulation, 3 drops of 5 μL phage stock solution were added and incubated at 37 °C overnight, and the next day, the transparent or cloudy lysis area was observed. The phage was concentrated and purified by PEG precipitation with reference to the method in the literature [27].

### 2.4. Analysis of Phage Morphology and Biological Characteristics

#### 2.4.1. Morphological Observation of Phage

A total of 20 μL purified phage was dropped on a 200-mesh carbon membrane copper mesh and placed there for 3–5 min, and excess liquid was removed by suction with filter paper. Then, 2% phosphotungstic acid was dropped and placed for 1–2 min, the excess liquid was absorbed, and then the phage was dried at room temperature and observed under the transmission electron microscope (TEM).

#### 2.4.2. Phage Host Spectrum Test

Double agar overlay plaque assay was used to determine the host spectrum of the phage, and the efficiency of plating (EOP) was calculated. The purified phage solution (10^9^ PFU/mL) was diluted to different concentration gradients (10–10^8^ PFU/mL) with SM buffer. A total of 100 μL of overnight-cultured MRSA bacteria solution was mixed with 100 μL of diluted phage solution and then cultured overnight with double agar overlay plaque assay. The plaque formation was observed and the number of plaques was counted (30–300 was appropriate).

#### 2.4.3. Determination of the Optimal Multiplicity of Infection (MOI)

Ten-fold gradient dilutions of phages were prepared with SM buffer at a MOI of 0.001, 0.01, 0.1, 1, 10, and 100. A total of 100 μL of the tested phage and 100 μL of the logarithmic phase MRSA 1 (10^7^ CFU/mL) were added to 6 mL liquid medium and incubated at 37 °C overnight. The culture was centrifuged at 4000 rpm for 15 min, and the supernatant was filtered through a 0.22 μm filter to remove the bacteria. The titer of the phage was determined by double plate method, and the MOI with the highest titer in each experimental group was the optimal MOI. Three parallel experiments were performed for each group.

#### 2.4.4. One-Step Growth Curve Test

A total of 10^9^ CFU/mL MRSA and phage were mixed at the optimal MOI of 0.01 and centrifuged at 15,000 rpm for 5 min after 37 °C incubation for 5 min. The supernatant was discarded, and 900 μL of liquid medium was added to resuspend the precipitate. The resuspension was added to 9 mL of liquid medium and incubated at 37 °C with shaking at 180 rpm. Samples were taken every 10 min and the titer of the phage was determined by double agar overlay plaque assay for 180 min. Three parallel experiments were performed for each group.

#### 2.4.5. Thermal and pH Stability Assays

A total of 1 mL of each phage (3 × 10^9^ PFU/mL) was placed in a 1.5 mL centrifuge tube and incubated at 4, 25, 37, 40, 50, 60, 70, 80, 90, and 100 °C for 1 h. Then, the phage titer was determined by the double agar overlay plaque assay. Three parallel experiments were performed for each group.

A total of 100 μL of each phage (3 × 10^9^ PFU/mL) was added to 900 μL of SM buffer at pH 2.0, 4.0, 6.0, 7.0, 8.0, 10.0, and 12.0. After incubation for 1 h at 37 °C, the phage titer was determined by the double agar overlay plaque assay. Three parallel experiments were performed for each group.

### 2.5. Analysis of Phage Genome

vB_SauS_PHM genome extraction and nucleic acid type identification were carried out according to the method reported by Ding et al. [28]. Guangzhou Huiyuanyuan Medical Technology Co., Ltd. (Guangzhou, China) was commissioned to perform paired-end (PE) sequencing using the second-generation sequencing Illumina sequencing platform. Open Reading Frames (ORFs) were identified through the RAST online server and the GeneMarks server. The functions of amino acid sequence proteins were predicted using the non-redundant (NR) protein database of Blastp online server. The genome information of vB_SauS_PHM was uploaded to NCBI and the GenBank number was OR206057. SnapGene v.5.0. was used to create a genome map.

The virulence factor and antibiotic resistance genes in vB_SauS_PHM genome were identified using the Comprehensive Antibiotic Resistance Database (CARD) and the virulence factor database (VFDB). Potential tRNA genes were identified using tRNA scan-SE v.2.0. Blastn online server was used for nucleotide sequence similarity identification and comparative analysis of full-length genome sequences. Phage nomenclature and classification were performed according to the guidelines of International Committee on the Taxonomy of Viruses (ICTV) and Bacterial and Archaeal Viruses Subcommittee (BAVS) [29]. The mafft v7.037 software was used for whole-genome sequence alignment, and IQTree v 1.6.12 software was used to construct a maximum likelihood phylogenetic tree with a bootstrap value of 1000.

### 2.6. Preparation and Characterization of Lip@PHM

#### 2.6.1. Preparation of Lip@PHM by Microfluidic Method

**Figure 1:**
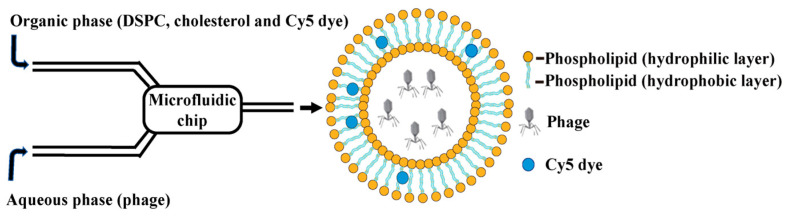
Schematic diagram of Lip@PHM prepared by microfluidic method (After the organic phase is mixed with the aqueous phase, DSPC will experience a highly polar environment, which will make DSPC self-assemble into spherical liposomes, thus causing phages in the surrounding aqueous phase to be captured into the hydrophilic layer inside the liposomes. In addition, cyanine dye Cy5 is hydrophobic and will be trapped into the hydrophobic layer inside liposomes).

Referring to methods in the [20,30] and improving upon them, Lip@PHM was prepared by the microfluidic method (Figure 1). The microfluidics equipment (type: Microflow S) was purchased from Mingtai pharmaceutical Equipment Co., Ltd. (Shanghai, China). The microfluidic chip was designed and fabricated by the Institute of Electronics, Chinese Academy of Sciences. The solution of 1,2-distearoyl -sn- glycerol -3-phosphorylcholine (DSPC) and cholesterol with a concentration of 20 mg/mL was prepared with ethanol, which was mixed to 0.6 mL at a ratio of 5: 1 and preheated to 60 °C. Then, 2 mg cyanine dye Cy5 was added, mixed thoroughly, and used as an organic-phase liquid dispersion. The aqueous phase was 1.8 mL vB_SauS_PHM (10^10^ PFU/mL) at room temperature. The organic-phase liquid dispersion was filtered through a 0.22 μm filter membrane. The organic-phase liquid dispersion was aspirated into a 1 mL syringe and the aqueous phase solution into a 3 mL syringe, and the air in the syringe was drained. The outlet of the syringe and the sample inlet tube were connected, and the flow rate of the organic phase was set to 0.3 mL/min, and the flow rate of the aqueous phase was set to 0.9 mL/min. After two minutes of mixing in the microfluidic chip, the temperature was about 35 °C. Samples were collected in 100 KDa dialysis bags and dialyzed with ultrapure water for three days, during which the water was changed every 12 h. The dialysate was collected and concentrated in an ultrafiltration tube. The purpose of dialysis liposome dispersion was to remove residual ethanol. The dialysis volume was 1 L of ultrapure water, which was changed every 12 h.

#### 2.6.2. Purification and Characterization of Lip@PHM

The unpurified Lip@PHM reaction liquid dispersion contained the remaining free phage and empty liposome, which differed in size from Lip@PHM and could be purified by size-exclusion chromatography. The size-exclusion chromatography column was purchased from Rengen Biosciences (Yingze Tonghui Biology Science and Technology Co., Ltd., Beijing, China) and packed with Sephadex G-50, length 30 cm. The size-exclusion chromatography column was removed from 4 °C and left at room temperature for at least 30 min. Activated column: The column bed was washed repeatedly more than 10 times by adding 5 mL ultrapure water and 15 mL PBS in turn. After loading 1 mL of the sample, the bottom cover was removed, and when the liquid was completely immersed in the column bed, 1 mL ultrapure water was added to collect the outflow liquid. The column was rinsed by adding 20 mL ultrapure water.

The hydrodynamic diameter and Zeta potential of Lip@PHM liquid dispersion before and after purification were measured using a nanoparticle size and Zeta potential analyzer according to the manufacturer’s instructions.

### 2.7. Preparation and Characterization of TAT-Lip@PHM

1, 2-distearyl-sn-glycerol-3-phosphate ethanolamine polyethylene glycol (DSPE-PEG_2000_) has amphiphilicity, low toxicity, and biodegradability. After combining with different biomolecules, it can functionalize the drug carrier to enhance circulation time and stability [31]. Preparation of TAT-PEG_2000_-DSPE: A total of 1 mmol DSPE-PEG_2000_ was dissolved in 200 μL boric acid buffer (pH = 8.4), and 0.1% EDTA and 1 mmol TAT peptide were added. TAT peptide was purchased from GuangZhou Tanshtech Co., Ltd; Guangzhou, China. (sequence: YGRKKRRQRRR). TAT-PEG_2000_-DSPE was synthesized via amide bond formation between the carboxyl group of DSPE-PEG_2000_ and the amine group of TAT peptide, catalyzed by EDTA in boric acid buffer (pH 8.4). The mixture was shaken overnight at room temperature at 180 rpm and then desalted by an exclusion column and separated by high-performance liquid chromatography (C18 column, water/acetonitrile with 0.1% TFA, 1 mL/min, detection at 220 nm). The peak product (TAT-PEG_2000_-DSPE) at 30 min was collected, lyophilized, and stored at −20 °C until use. Lip@PHM was incubated with 1 μM TAT-PEG_2000_-DSPE at 37 °C for 1 h, and then excess TAT-PEG_2000_-DSPE was removed using a 100 KDa ultrafiltration tube (Winter song Boye Biotechnology Co., Ltd., Beijing, China) to obtain TAT-Lip@PHM, which was stored at 4 °C until use.

The hydrodynamic diameter and Zeta potential of TAT-Lip@PHM were measured using the nanoparticle size and Zeta potential analyzer, as described above. The medium for measuring the zeta potential of all samples is PBS (pH 7.4).

The phage encapsulation efficiency of TAT-Lip@PHM was determined according to the reported method [20]: After the demulsification of TAT-Lip@PHM through 0.5% Triton X-100, 100 μL TAT-Lip@PHM and 900 μL of 0.5% Triton X-100 were mixed in a 1.5 mL centrifuge tube and left at room temperature for 30 min. The phage titer was counted by double agar overlay plaque assay. Encapsulation efficiency (%) = number of encapsulated phages/total number of phages ×100%. Three parallel experiments were performed for each group.

The fluorescence intensity of TAT-Lip@PHM was detected using a fluorescence spectrophotometer with emission wavelengths of 670–860 nm.

### 2.8. Cytotoxicity Assay of TAT-Lip@PHM

MTT assay was used to determine the toxicity of different concentrations of TAT-Lip@PHM on RAW 264.7 mouse monocytes and macrophages. The adherent RAW 264.7 cells were resuspended in complete medium, seeded into 96-well plates at a density of 100 μL per well and 3000–10,000 cells/well, and cultured overnight. After the cells were attached to the wall, 100 μL TAT-Lip@PHM was added at final concentrations of 0, 150, 300, 600, and 1200 μg/mL, respectively, and three parallel wells were set for each concentration. Then, the cells were cultured at 37 °C in a 5% CO_2_ cell incubator for 24 h. The liquid in the wells was removed, and the cells were slowly rinsed three times with PBS. A total of 90 µL complete medium and 10 µL MTT solution were added to each well. After 4 h of incubation, the liquid in the wells was aspirated and 110 µL formazan solution was added to each well. The microplate was placed on the oscillator and shaken at 300 rpm for 10 min to dissolve all the crystals. The OD_490_ value was measured by microplate reader, and the cell survival rate was calculated as (OD value of experimental group − OD value of blank group)/(OD value of control group − OD value of blank group) × 100%.

### 2.9. Intracellular Localization of TAT-Lip@PHM

Adherent RAW 264.7 cells were resuspended in complete medium, and 1.5 mL of the cells were seeded into a confocal culture dish at a density of about 10^6^ cells/mL. TAT-Lip@PHM was added to the culture dish with a total lipid concentration of 300 μg/mL (containing ~3.7 × 10^8^ PFU/mL phage). The fluorescence status was observed under confocal microscope at 2, 4, 6, 12, and 24 h, and the proportion of cells was calculated as the number of fluorescent cells/the total number of cells.

### 2.10. Eradication Effect of TAT-Lip@PHM on Intracellular MRSA Infection

#### 2.10.1. Construction of an Intracellular MRSA Infection Cell Model

The intracellular bacterial model was constructed by infecting RAW 264.7 cells with MRSA strain. The adherent cells were resuspended in complete medium and seeded into six-well plates at a density of 1.02 × 10^6^ cells/mL. After the cell adherence, about 2 × 10^7^ CFU/mL of MRSA in complete medium was mixed with the cells, that is, 10–20 bacteria/cell. After 2 h of incubation, the culture medium was discarded and slowly rinsed with PBS buffer three times, and then the culture medium containing 200 μg/mL gentamicin was cultured for 4 h. The culture medium was discarded and rinsed with 1 mL PBS buffer three times, and the intracellular bacteria were filmed using an inverted fluorescence microscope. After adding 2 mL PBS buffer, the plate colony-counting method was used to count the extracellular bacteria. PBS was aspirated, and 2 mL of 0.5%Triton X-100 was added to lyse the cells, which were incubated at room temperature for 30 min and blown. The intracellular bacterial concentration was counted by the flat colony counting method. Three parallel experiments were performed for each group.

#### 2.10.2. Evaluation of Intracellular MRSA Eradication

The cells infected with intracellular MRSA were replaced with 3 mL of complete culture medium, and three experimental groups were treated with the addition of 1 mL of vB_SauS_PHM (final concentration of 1.5 × 10^9^ PFU/mL), 1 mL of Lip@PHM (300 µg/mL, final concentration of phage at 5.4 × 10^8^ PFU/mL), and 1 mL of TAT-Lip@PHM (300 µg/mL, final concentration of phage at 3.7 × 10^8^ PFU/mL), respectively. For the control group, 1 mL of PBS buffer was added. After each group was cultured for 12, 18, and 24 h, the liquid in the well plate was aspirated and slowly rinsed with PBS; then, 2 mL 0.5%Triton X-100 was added to lyse the cells, the cells were treated for 30 min, and the intracellular bacteria were counted. The intracellular MRSA clearance rate was calculated according to the following formula:

Clearance rate = (number of intracellular bacteria in PBS control − number of intracellular bacteria in experimental group)/number of intracellular bacteria in control group × 100%. Three parallel experiments were performed for each group.

## 3. Results

### 3.1. Biological Characteristics of Phage vB_SauS_PHM

**Figure 2:**
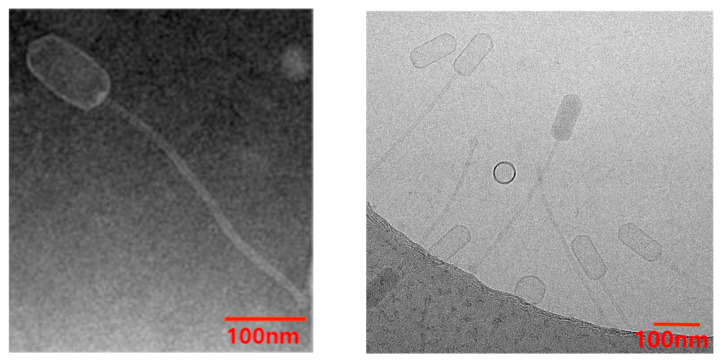
TEM images of phage vB_SauS_PHM.

In this study, a clinical isolate named MRSA 1 was used as the host bacteria to isolate a new phage named Staphylococcus phage vB_SauS_PHM from the sewage of a hospital in Beijing (NCBI registration No. OR206057, vB_SauS_PHM for short). vB_SauS_PHM could make the bacterial lawn of MRSA 1 appear as a transparent lysis region (Figure S1A); the plaque diameter was about 0.5 mm, the size was uniform, and the edge was neat, without a halo (Figure S1B). The phage titer reached 10^10^ PFU/mL after concentration by the PEG precipitation method. As observed by TEM (Figure 2), vB_SauS_PHM consists of an ellipsoid head (~100 nm × 50 nm) and a long tail (~310 nm) with a a non-retractable tailless sheath. vB_SauS_PHM was similar to the phage JD419 (NCBI sequence No. MT899504.1) [32], belonging to *Duplodnaviria*, *Caudovirales*, and *Siphoviridae*.

**Figure 3:**
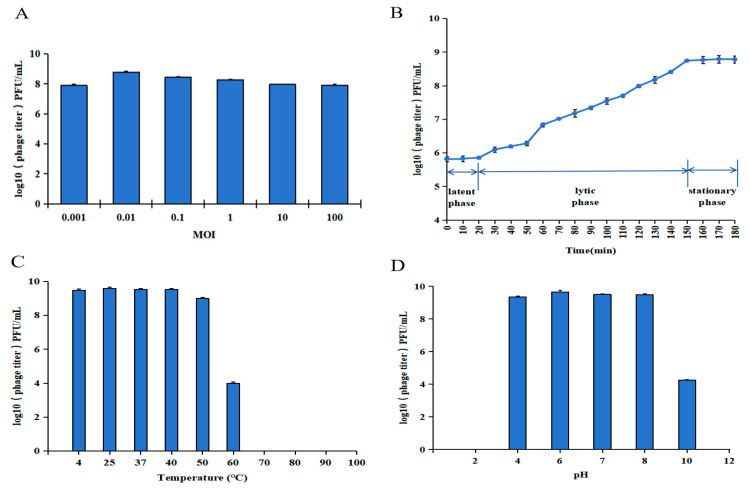
Biological characteristics of vB_SauS_PHM. The optimal multiplicity of infection (**A**). One-step growth curve (**B**). Thermal stability (**C**). pH stability (**D**).

The lysis spectrum of vB_SauS_PHM was measured using 12 clinical MRSA strains of different origins, as shown in Table S1, and the lysis rate was 67% (8/12). As shown in Figure 3A, when the MOI was 0.01, vB_SauS_PHM had the highest titer of 6.2 × 10^8^ PFU/mL, indicating that the optimal MOI was 0.01. The one-step growth curve of vB_SauS_PHM is shown in Figure 3B. The incubation period of vB_SauS_PHM was 20 min, the lysis period was 130 min, and the lysis amount was about 119.4 PFU/cell; it entered the stable phase after 150 min.

The thermal stability of vB_SauS_PHM was evaluated by measuring the titer of the phage at different temperatures. As shown in Figure 3C, the titer of vB_SauS_PHM was stable (~10^9^ PFU/mL) at 4–40 °C. The titer decreased by about 1 log and 5 log at 50 °C and 60 °C, respectively, and the titer was 0 at ≥70 °C, indicating that vB_SauS_PHM was most stable at 4–40 °C. The pH stability of vB_SauS_PHM is shown in Figure 3D. The titer of vB_SauS_PHM was stable at pH 4–8, at about 10^9^ PFU/mL. When the pH was 10, the titer of the phage decreased significantly by about 5 log. And when pH ≥ 12 or pH ≤ 2, the phage titer was 0.

### 3.2. Genome Analysis of Bacteriophage

The result of nucleic acid electrophoresis of the vB_SauS_PHM genome is shown in Figure S2. The genome could be completely digested by DNase I, but not by Rnase A and Mung Bean Nuclease, indicating that the genome of vB_SauS_PHM was double-stranded DNA.

**Figure 4:**
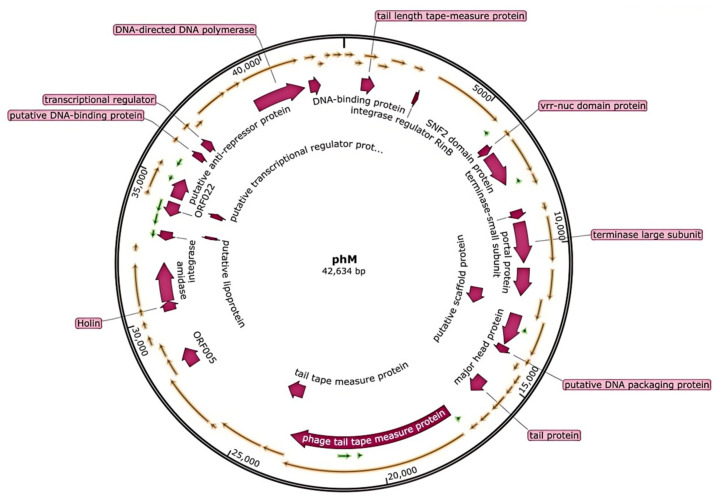
Genome map of vB_SauS_PHM. Note: The arrows indicate the direction of transcription of each gene.

The genome map of vB_SauS_PHM is shown in Figure 4. It was 42,634 bp in length (33.79% G/C) and contained 59 Open Reading Frame (ORF) groups, of which 23 ORFs were predicted to encode functional proteins (Table S2) with no putative tRNA genes. The genome of vB_SauS_PHM did not contain known virulence factors or antibiotic resistance genes as checked by VFDB and CARD. Phylogenetic relationships between vB_SauS_PHM and other staphylococcal phages are shown in Figure S3; the most closely related are bacteriophages SAP11 (MK801681.1) and SMSAP5 (NC_019513.1, JQ779023.1). According to ICTV guidelines and morphological characteristics [29], vB_SauS_PHM belonged to *Duplodnaviria*, *Caudovirales*, *Siphoviridae*, and *Triavirus*.

### 3.3. The Characterization Results of Lip@PHM

The characterization of all the measured samples is shown in Table 1. The preparation of Lip@PHM was conducted using microfluidic assembly, followed by purification through SEC. As shown in Table 1, the zeta potential of the Lip@PHM stock liquid dispersion, generated by microfluidic assembly, exhibited a positive shift (from −10.4 ± 0.9 mV to −2.1 ± 0.6 mV) compared to that of the component liquid dispersion prior to assembly. This shift could be attributed to the encapsulation of the negatively charged phage (Zeta potential: −13.7 ± 0.9 mV) by the liposomes after self-assembly, generating the neutrally charged Lip@PHM. This also indicated the successful preparation of Lip@PHM. Then, the Lip@PHM was purified through SEC, resulting in a Zeta potential of −0.7 ± 0.1 mV, which was more neutral compared to the pre-purification stock liquid dispersion. This was due to the fact that the purification process removed the free phage. Furthermore, the particle size distribution of Lip@PHM after purification exhibited greater uniformity compared to the stock liquid dispersion prior to purification, with the PDI changing from 0.197 ± 0.019 to 0.136 ± 0.009.

### 3.4. Characterization of TAT-Lip@PHM

TAT-Lip@PHM, prepared using the TAT modification of Lip@PHM, had an average hydrodynamic diameter of 637.7 ± 5.7 nm (PDI:0.146 ± 0.010) and a zeta potential of 3.9 ± 0.2 mV (Table 1), showing a slight increase in hydrodynamic diameter compared to that of Lip@PHM, and the zeta potential was reversed from negative to positive. This is due to the modification of TAT on the surface of the composite to increase its particle size; at the same time, TAT is a strong cationic polymer, which reversed the potential of the composite, indicating that we have successfully prepared the TAT-Lip@PHM delivery system.

The ability to carry “cargo” is an important indicator of the performance of the carrier system, and we determined the encapsulation rate of the phage by TAT-Lip@PHM. When 1.8 mL of free phage with a titer of 10^10^ PFU/mL was added, the amount of TAT-Lip@PHM prepared was 5 mL, and the titer of the encapsulated phage (measured after demulsification) was 7.3 × 10^8^ PFU/mL. The encapsulation efficiency of the phage by TAT-Lip@PHM was 20.3% (7.3 × 10^8^×5/1.8 × 10^10^ = 20.3%).

### 3.5. The Biological Activity of TAT-Lip@PHM

**Figure 5:**
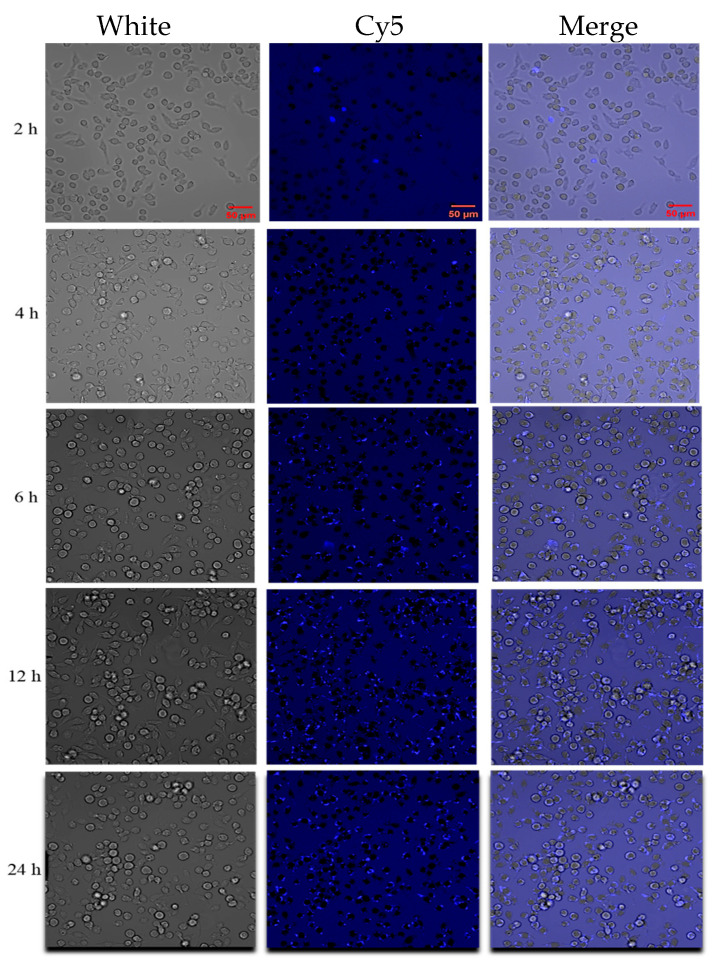
Laser confocal microscopy images of TAT-Lip@PHM ingested by mouse monocyte macrophage RAW 264.7 at varying time points (scales were 50 μm).

For intracellular localization, the Cy5 dye was encapsulated using TAT-Lip@PHM. Following encapsulation, the peak emission wavelength of TAT-Lip@PHM was observed to be approximately 690 nm (as illustrated in Figure S5), which was consistent with the Cy5 dye, indicating that the Cy5 dye had been successfully encapsulated within the liposome. The fluorescence microscopy images of TAT-Lip@PHM following cellular uptake at varying time points are presented in Figure 5. The number of intracellular fluorescent dots increased in accordance with the duration of incubation, indicating that the uptake of TAT-Lip@PHM by the cells was gradually enhanced. Following incubation periods of 2, 4, 6, 12, and 24 h, the proportion of TAT-Lip@PHM internalized by cells was 5.33%, 43.30%, 67.37%, 92.98%, and 94.38%, respectively. The cellular uptake reached a level exceeding 90% at 12 h of incubation, indicating that the majority of TAT-Lip@PHM was taken up at approximately 12 h.

**Figure 6:**
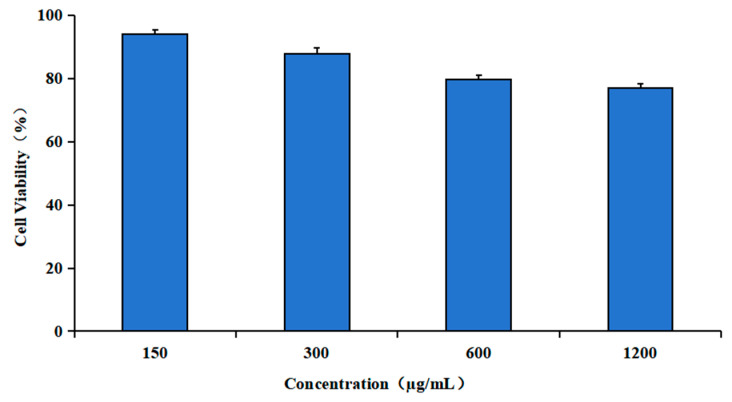
Viability of RAW 264.7 cells treated with different final concentrations of TAT-Lip@PHM for 24 h.

The biotoxicity of a drug carrier represents a significant factor influencing its potential applications. To investigate the toxicity of varying concentrations of TAT-Lip@PHM on RAW 264.7 cell, we employed the MTT assay. The results, as illustrated in Figure 6, demonstrated that following the co-incubation of RAW 264.7 cells with TAT-Lip@PHM at final concentrations of 150, 300, 600, and 1200 µg/mL for 24 h, the cellular activities were 94.19%, 87.98%, 79.84%, and 77.13%, respectively. These findings indicated that TAT-Lip@PHM at 300 µg/mL and below exhibited good cytocompatibility, with >85% cell viability.

**Figure 7:**
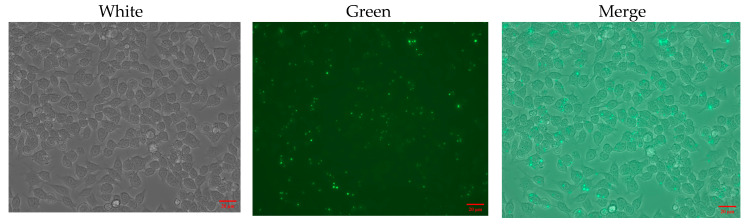
Inverted fluorescence microscope of RAW 264.7 cells ingesting green fluorescent-labeled MRSA strain after 2 h of incubation.

In order to evaluate the effect of phage-delivered composites on intracellular MRSA clearance, an intracellular MRSA-infected cell model was constructed. As illustrated in Figure 7, following the co-culture of cells with MRSA under antibiotic pressure, green fluorescently labeled MRSA strains were discernible under fluorescence microscopy as intracellular, with no fluorescent bacteria observed outside the cells. The number of extracellular and intracellular bacteria was determined by plate culture (Figure S6). No colonies were observed outside the cells, and the number of intracellular bacteria was 3 × 10⁶ CFU, indicating that the cell model of intracellular MRSA infection was successfully constructed.

**Figure 8:**
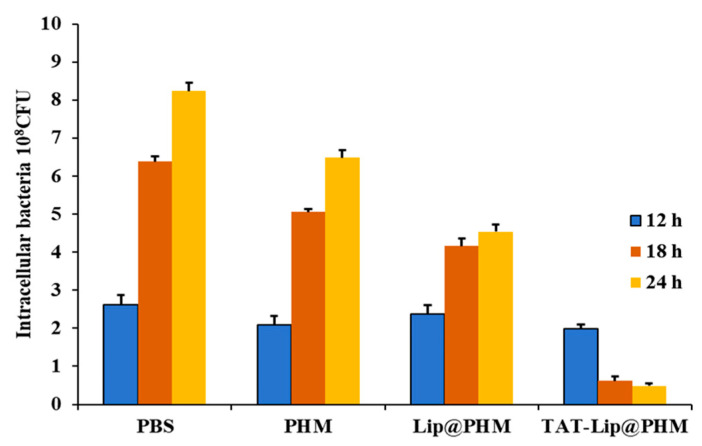
Plate colony count of intracellular MRSA after different times of TAT-Lip@PHM treatment.

The phage vB_SauS_PHM (1.5 × 10^9^ PFU/mL), Lip@PHM (containing ~5.4 × 10^8^ PFU/mL phage) and TAT-Lip@PHM (~3.7 × 10^8^ PFU/mL phage) were employed to treat intracellular MRSA-infected cells in a series of experiments. To evaluate the clearance effect of the different materials on intracellular MRSA, the number of intracellular colonies in each group was counted at 12, 18, and 24 h. The results are presented in Figure 8 and Figure S7. A comparison of the experimental groups with the PBS control group revealed a reduction in the number of intracellular bacteria. The intracellular MRSA clearance rates of the vB_SauS_PHM and Lip@PHM groups were 21.24% and 44.90%, respectively. The Lip@PHM group exhibited a slight improvement over the phage group, which can be attributed to the inherent capacity of liposomes to facilitate the internalization of phages into cells and provide a degree of protection for their intracellular activity. In contrast, the TAT-Lip@PHM group exhibited the lowest number of intracellular MRSA colonies among all groups at each time point, thereby demonstrating excellent bactericidal ability. It was evident that despite the relatively low concentration of the phage in the TAT-Lip@PHM group, it demonstrated the most effective clearance of intracellular MRSA (94.05% at 24 h). The encapsulation of liposomes and the modification of TAT appeared to exert a systematic effect, with the liposomes primarily serving as a protective vehicle for the phage, while the modification of TAT further facilitated the ability of the carrier composite to penetrate into the cell interior, thereby facilitating the killing of intracellular bacteria by phages.

## 4. Discussion and Conclusions

Though lots of studies have employed drug carrier systems to treat intracellular MRSA infections, the majority of drugs encapsulated were antibiotics, including rifampicin, penicillin, and vancomycin [33,34,35,36]. In comparison to antibiotics, phages possess several advantages, including high specificity [37,38], low toxicity [39,40], low development cost [41], and a relatively low propensity for resistance [42]. Phage vB_SauS_PHM was isolated from hospital effluent and demonstrated the capacity to lyse multiple clinical MRSA strains (8/12), exhibiting a superior lysis spectrum to that of the reported *S. aureus* phage vB_SauM_ME18 [43]. vB_SauS_PHM had an MOI of 0.01, a short latency of 20 min, and a lysing amount of 119.4 PFU/cell. It exhibited a shorter latency and greater lysis capacity than that (a latency of 50 min and a lysing amount of 33 PFU/cell) of the mild phage JD419 reported by Feng et al. [32]. Furthermore, the bactericidal efficacy of phages is also influenced by their environmental tolerance. The vB_SauS_PHM phage was found to be potent and stable at temperatures ranging from 4 to 40 °C and pH levels between 4 and 8. This indicates that the vB_SauS_PHM phage has good environmental tolerance. The temperature stability of vB_SauS_PHM was superior to that of *S. aureus* phages vB_SauM_JDYN, as reported by Guo et al., and MSP, as reported by Ganaie et al. [44,45]. Additionally, its acid resistance was more pronounced than that of phage VW (NCBI sequence no.: MT787017) [46], further underscoring the exceptional characteristics of vB_SauS_PHM.

The current research on the utilization of phages for the treatment of intracellular MRSA is limited and still in its infancy [47]. Bispo et al. [48] coupled a phage’s endolysin cell-binding domain with light-activated materials. The resulting coupling material was observed to be specifically internalized by MRSA-infected cells and demonstrated a notable targeting ability for the eradication of intracellular MRSA upon light activation. However, the principal bactericidal agent in their study was a photosensitive material, which necessitated the use of additional photogeneration apparatus and was therefore unable to be effective in deep infection due to the limitation of light penetration. Wang et al. [25] combined phage lyase with the cell-penetrating peptide TAT, which enhanced the ability of the composite material to enter the cell and achieved a clearance rate of 80% for MRSA in keratinocytes.

The TAT-Lip@PHM delivery system prepared in this study combines the specific bactericidal property of phage, the good ability of liposomes to transport and protect “cargo”, and the ability of TAT to penetrate the cellular barrier. TAT-Lip@PHM demonstrated an efficient delivery of the phage into the cell (≥90% at 8 h) and an effective clearance of intracellular MRSA (94.05% at 24 h). TAT-Lip@PHM demonstrated a stronger clearance effect on intracellular MRSA with a reduced phage concentration in comparison to free phage, while also exhibiting favorable cytocompatibility.

We observed that the free phage alone performed better than the PBS control. We attribute it to the fact that phage is intracellularized through non-specific micropinocytosis. The cellular uptake of phages was first observed in professional phagocytes, such as macrophages or granulocytes. For this reason, it was historically referred to as “phagocytosis”. The modern definition of phagocytosis, however, identifies this process as a type of endocytosis within a larger repertoire of endocytic pathways, such as macropinocytosis, clathrin-mediated endocytosis, and caveolar endocytosis. Among those, phagocytosis and macropinocytosis apply to objects measured in micrometers (e.g., bacteria), so they seem highly applicable for engulfment of phages, including the largest ones (Caudovirales) [49]. According to “Section 3.2”, the phage (vB_SauS_PHM) in this study belongs to Duplodnaviria, Caudovirales. In addition, the cells used to construct the intracellular MRSA-infected cell model in this study were RAW 264.7 mouse mononuclear macrophages.

In addition, we further discussed the possible mechanism of the internalization of TAT-Lip@PHM and the release of the phage from the liposomes. At present, most liposomes are transported by lysosomes after being ingested by cells. In this process, bacteriophages in liposomes are easily inactivated by enzymes, thus reducing the intracellular concentration and bactericidal efficacy. In order to prevent this phenomenon, the modified cell-penetrating peptide (TAT peptide) is used to give liposomes the ability to escape or escape lysosomes and enter cells through the direct penetrating route independent of energy, thus bypassing the lysosomal degradation process in the endocytosis route, achieving the degradation in the cytoplasm and releasing phages [21,22,50].

Furthermore, in order to solve the problem that liposomes prepared by traditional methods such as electroformation [51,52], thin-film hydration [53] and sonication [54] cannot encapsulate large-size vB_SauS_PHM (~410 nm) [19], we adopted a new microfluidic method to produce large single-compartment liposomes. By optimizing the material ratio and production parameters, large single-compartment liposomes with sizes up to ~600 nm were prepared, and they were capable of encapsulating the vB_SauS_PHM with an encapsulation rate of 20.3%.

In conclusion, this study demonstrated an effective approach for the eradication of intracellular drug-resistant bacteria utilizing cell-penetrating peptide-modified liposomes to facilitate the delivery of the phage into the cell. It would be a promising strategy against the rising tide of antibiotic resistance and the difficulties associated with treating intracellular bacterial infections. This study could also provide a technical reference for the application of phage to treat intracellular drug-resistant bacterial infections.

## Figures and Tables

**Table 1:** Characterization of all the measured samples (*n* = 3, mean ± SD).

Sample	Hydrodynamic Diameter (nm)	Polydispersity Index (PDI)	Zeta Potential (mV)
Phage vB_SauS_PHM	234.3 ± 7.5	0.217 ± 0.005	−13.7 ± 0.9
Mixture of lipids and phages without microfluidic assembly	double peaks	——	−10.4 ± 0.9
Lip@PHM before purification	577 ± 5.3	0.197 ± 0.019	−2.1 ± 0.6
Lip@PHM after purification	627.7 ± 5.0	0.136 ± 0.009	−0.7 ± 0.1
TAT-Lip@PHM	637.7 ± 5.7	0.146 ± 0.010	3.9 ± 0.2

## Data Availability

The data supporting this work are accessible upon reasonable request from the corresponding author.

## Supplementary Materials

The following supporting information can be downloaded at https://www.mdpi.com/article/10.3390/pharmaceutics17060743/s1, Figure S1: The lysis region (A) and plaques (B) of phage vB_SauS_PHM; Figure S2: Identification results of nucleic acid type of phage vB_SauS_PHM genome; Figure S3: Phylogenetic tree of the complete genome sequence of phage vB_SauS_PHM; Figure S4: Hydrodynamic diameter of Lip@PHM. (A) stock liquid dispersion before purification; (B) liquid dispersion after purification; Figure S5: Fluorescence emission spectrum of TAT-Lip@PHM; Figure S6: Plate colony count of intracellular MRSA infected cell model; Figure S7: Plate colony count of intracellular MRSA after treatment with TAT-Lip@PHM at different time. Table S1: The lysis spectrum of vB_SauS_PHM; Table S2: Predicted functional ORFs of phage vB_SauS_PHM.
