# Supplementary material for: A TAT Peptide-Functionalized Liposome Delivery Phage System (TAT-Lip@PHM) for an Enhanced Eradication of Intracellular MRSA
Source: Pharmaceutics. 2025 Jun 5;17(6):743. doi: 10.3390/pharmaceutics17060743 (PMC12196269; doi:10.3390/pharmaceutics17060743)
Supplement: Supplementary file 1 [file pharmaceutics-17-00743-s001.zip › pharmaceutics-3566099-supplementary.pdf]

SUPPORTING INFORMATION

A TAT peptide-functionalized liposome delivery phage system (TAT-Lip@PHM) for enhanced eradication of intracellular MRSA

Kaixin Liu [1†], Xin Lu[2†], Xudong Guo[1], Yi Yang[1], Wangying Liu[1], Hongbin Song[1]*, Rongtao Zhao[1]*

[1]Chinese PLA Center for Disease Control and Prevention, Beijing 100071, China

[2]The Fifth Medical Center of Chinese PLA General Hospital, Beijing 100071, China

[†]These authors contributed equally to the work.

*Corresponding author:

Rongtao Zhao, Email: zhaorongtao1984@163.com;

Hongbin Song, Email: hongbinsong@263.net

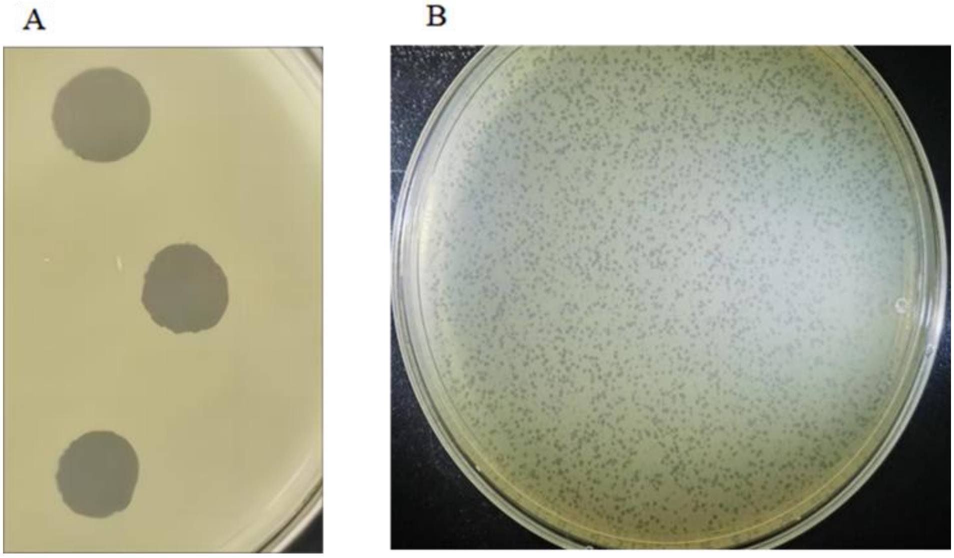

Figure S1. The lysis region(A) and plaques(B) of phage vB_SauS_PHM

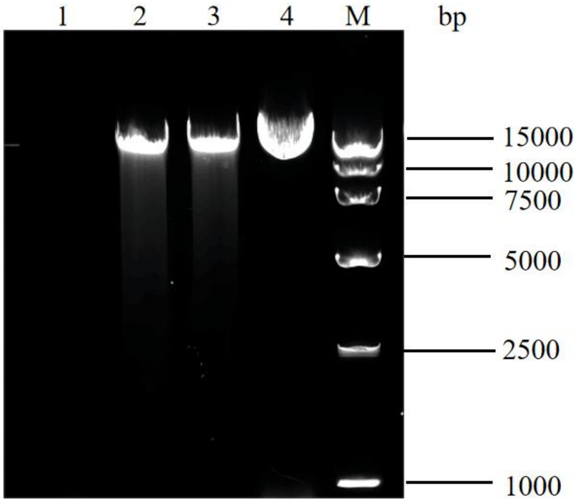

Figure S2. Identification results of nucleic acid type of phage vB_SauS_PHM genome. Note: 1. DNase I; 2. RNase A; 3. Mung bean nuclease; 4. Positive control; M. DNA Marker.

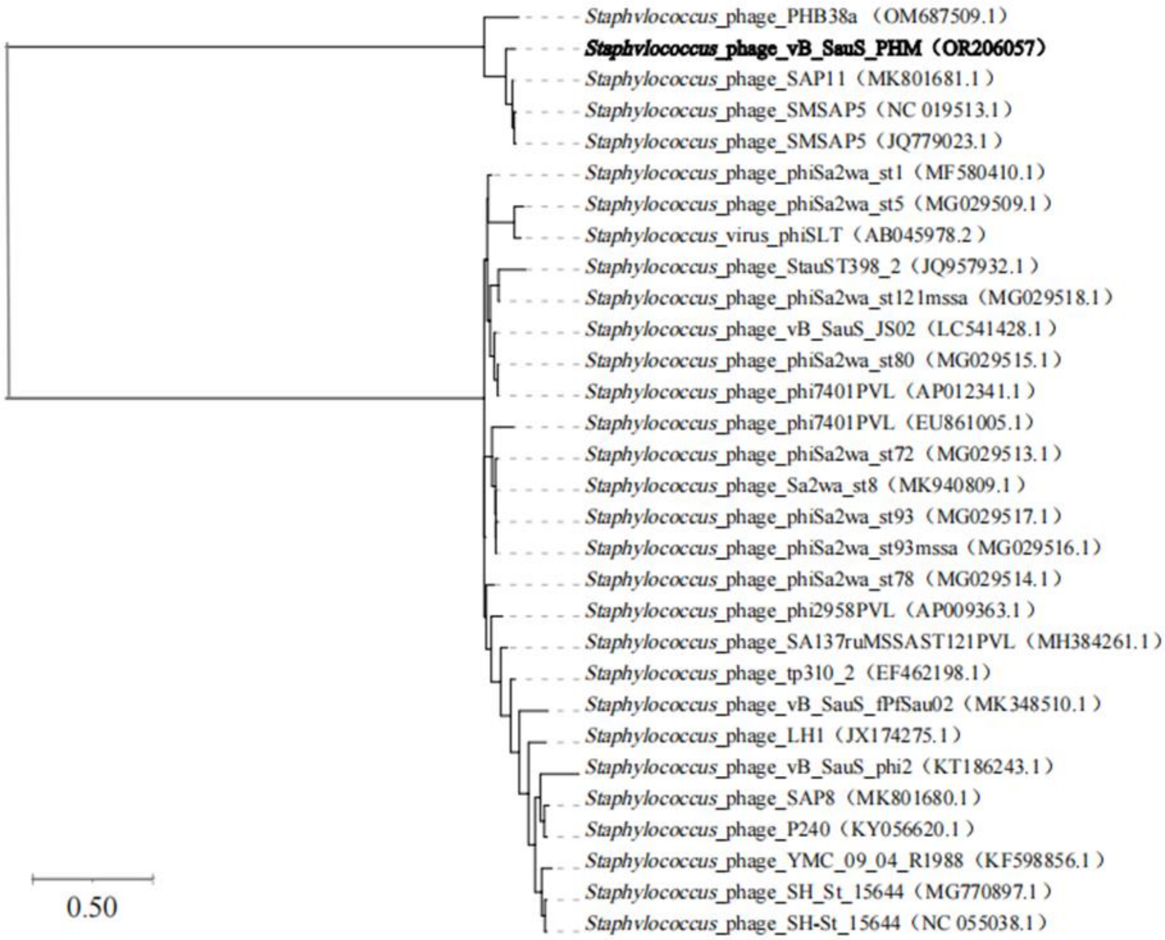

Figure S3. Phylogenetic tree of the complete genome sequence of phage vB_SauS_PHM. The numbers in parentheses represent GenBank sequence numbers, while the scales represent genetic distances.

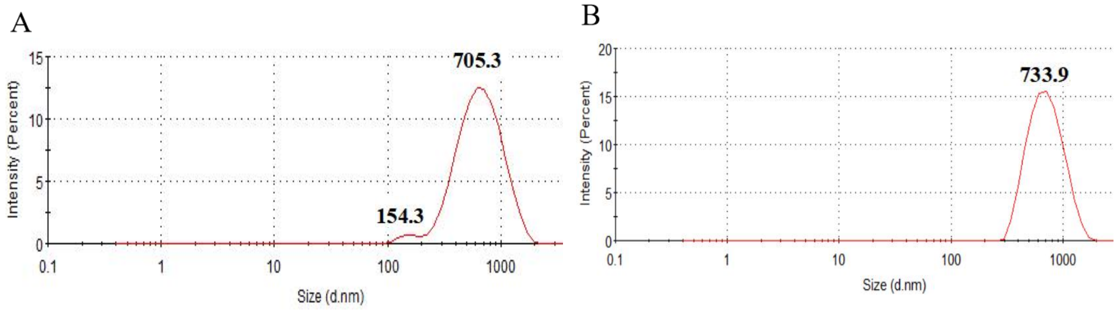

Figure S4. Hydrodynamic diameter of Lip@PHM. (A) stock liquid dispersion before purification; (B) liquid dispersion after purification.

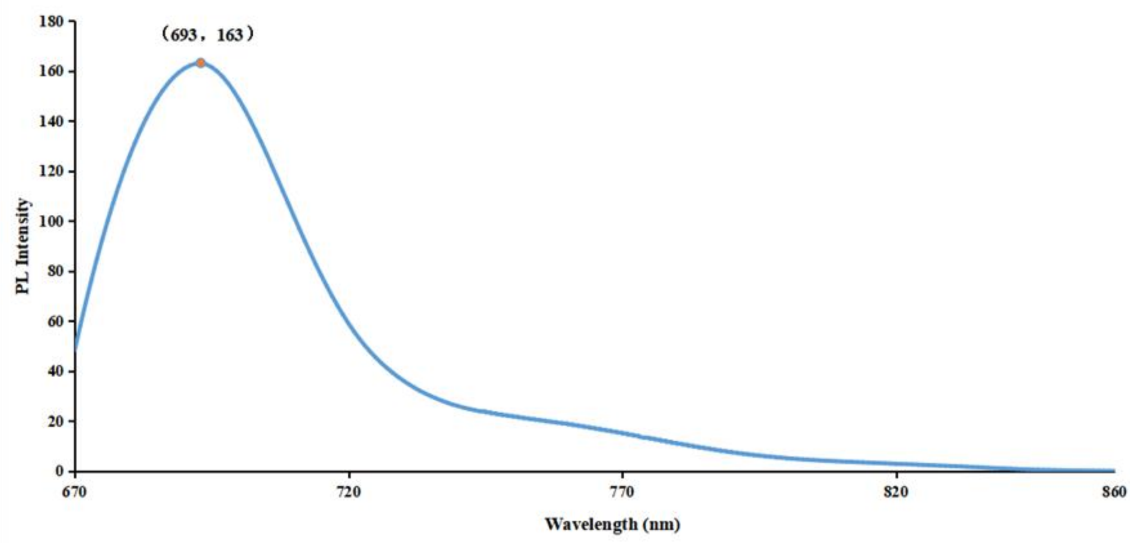

Figure S5. Fluorescence emission spectrum of TAT-Lip@PHM.

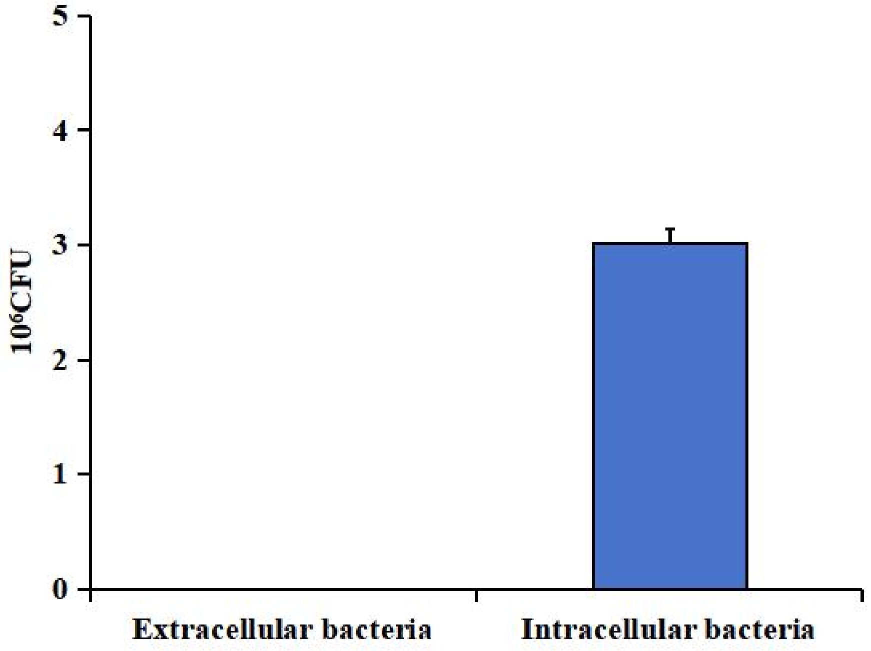

Figure S6. Plate colony count of intracellular MRSA infected cell model.

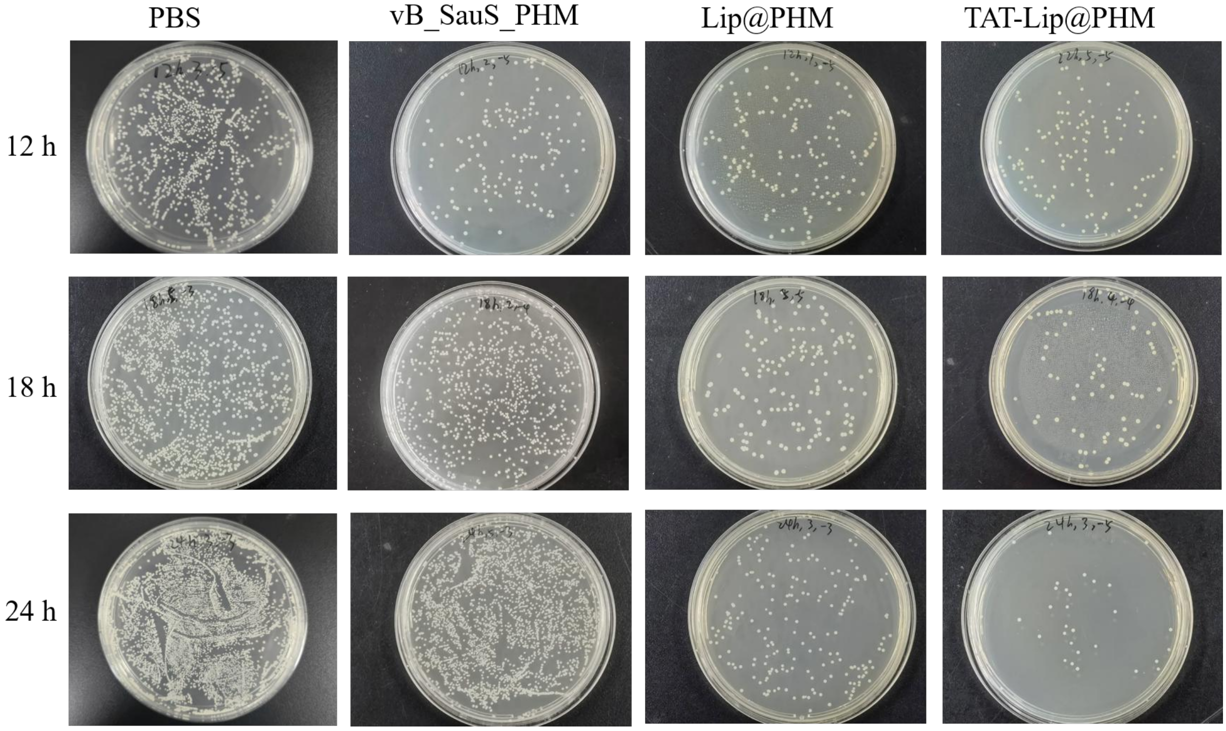

Figure S7. Plate colony count of intracellular MRSA after treatment with TAT-Lip@PHM at different time.

Table S1. The lysis spectrum of vB_SauS_PHM

Bacterial strains	Efficiency of plating
MRSA 1*	1
MRSA 2	-
MRSA 3	8.45×10^{-1}
MRSA 4	8.97×10^{-1}
MRSA 5	8.97×10^{-1}
MRSA 6	2.59×10^{-7}
MRSA 7	2.76×10^{-5}
MRSA 8	2.93×10^{-4}
MRSA 9	-
MRSA 10	-
MRSA 11	3.45×10^{-4}
MRSA 12	-

Note: "-": no phage plaque; "*": original host bacterium MRSA 1; EOP= titer of phage to test strain/titer of this phage to host bacterium MRSA 1.

Table S2. Predicted functional ORFs of phage vB_SauS_PHM

ORFs	Start	Stop	Length (bp)	Predicated function
ORF3	670	1155	486	tail length tape-measure protein
ORF8	2714	2866	153	integrase regulator RinB
ORF11	5974	6264	291	vrr-nuc domain protein
ORF12	6254	7612	1359	SNF2 domain protein
ORF15	8664	8969	306	terminase-small subunit
ORF16	8959	10650	1692	terminase large subunit
ORF17	10655	11893	1239	portal protein
ORF18	11877	12650	774	putative scaffold protein
ORF19	12662	13825	1164	major head protein
ORF20	13894	14172	279	putative DNA packaging protein
ORF24	15345	15986	642	tail protein
ORF28	17155	23355	6201	phage tail tape measure protein
ORF29	23355	24179	825	tail tape measure protein
ORF38	30227	30529	303	holin
ORF39	30541	31995	1455	lysin（N-acetylmuramoyl-L-alanine amidase）
ORF41	32842	33174	333	integrase
ORF42	33161	33280	120	putative lipoprotein
ORF45	34237	34473	237	putative transcriptional regulator protein
ORF46	34487	35263	777	putative anti-repressor protein
ORF50	36166	36429	264	putative DNA-binding protein
ORF51	36681	37004	324	transcriptional regulator
ORF55	39187	41148	1962	DNA-directed DNA polymerase
ORF57	41346	41747	402	DNA-binding protein
